# Impact of specialist and primary care stop smoking support on socio‐economic inequalities in cessation in the United Kingdom: a systematic review and national equity initial review completed 22 January 2019; final version accepted 19 July 2019 analysis

**DOI:** 10.1111/add.14760

**Published:** 2019-09-10

**Authors:** Caroline E. Smith, Sarah E. Hill, Amanda Amos

**Affiliations:** ^1^ GRIT, Usher Institute of Population Health Sciences and Informatics University of Edinburgh Edinburgh UK; ^2^ GRIT, Global Public Health Unit University of Edinburgh Edinburgh UK

**Keywords:** Cessation, disadvantage, primary care, smoking, socio‐economic inequalities, systematic review

## Abstract

**Aim:**

To assess the impact of UK specialist and primary care‐based stop smoking support on socio‐economic inequalities in cessation.

**Methods:**

Systematic review and narrative synthesis, with a national equity analysis of stop smoking services (SSS). Ten bibliographic databases were searched for studies of any design, published since 2012, which evaluated specialist or primary care‐based stop smoking support by socio‐economic status (SES) or within a disadvantaged group. Studies could report on any cessation‐related outcome. National Statistics were combined to estimate population‐level SSS reach and impact among all smokers by SES. Overall, we included 27 published studies and three collated, national SSS reports for England, Scotland and Northern Ireland (equivalent data for Wales were unavailable).

**Results:**

Primary care providers and SSS in the United Kingdom were particularly effective at engaging and supporting disadvantaged smokers. Low SES groups were more likely to have their smoking status assessed, to receive general practitioner brief cessation advice/SSS referral and to attempt a quit with SSS support. Although disadvantaged SSS clients were less successful in quitting, increased service reach offset these lower quit rates, resulting in higher service impact among smokers from low SES groups. Interventions that offer tailored and targeted support have the potential to improve quit outcomes among disadvantaged smokers.

**Conclusions:**

Equity‐orientated stop smoking support can compensate for lower quit rates among disadvantaged smokers through the use of equity‐based performance targets, provision of targeted services and the development of tailored interventions.

## Introduction

Throughout high‐income countries, inequalities in smoking contribute substantially to the unequal distribution of health by socio‐economic status (SES) 1. The need to reduce smoking among disadvantaged groups is therefore at the heart of UK tobacco control strategies 2, 3, 4, 5. As the only country in the world to have developed a national state‐funded system of cessation support, the United Kingdom provides a unique source of evidence on the effectiveness of such systems in tackling smoking inequalities.

Smokers in the United Kingdom who want to quit are able to access evidence‐based pharmacotherapy and behavioural support (delivered by specialist or community practitioners in a one‐to‐one or group format) through a network of stop smoking services (SSS) located in multiple settings, including pharmacies, general practitioner (GP) surgeries, community centres and work‐places 6. While these services are known to be effective in supporting cessation 7, systematic reviews consistently suggest an equity‐negative effect, with low SES service users having poorer quit rates than high SES users 7, 8, 9. Performance measures that encompass all smokers (not just SSS users), however, reveal a different picture. Bauld *et al*. 10, for instance, additionally examined SSS reach (the proportion of smokers making an SSS‐supported quit attempt) and SSS impact (the proportion of smokers making a successful SSS‐supported quit attempt). They found that, while quit rates were lower in more compared to less deprived areas (52.6 versus 57.9%), SSS reach was higher (16.7 versus 13.4%), giving an overall equity‐positive effect in relation to SSS impact (8.8 versus 7.8%). A systematic review by Brown *et al*. 8, moreover, reported similar findings, concluding that SSS can help to reduce smoking inequalities through the successful targeting and recruitment of disadvantaged smokers.

Alongside the SSS, the National Institute for Health and Care Excellence (NICE, the UK body responsible for national health‐care guidance) recommends that health professionals should seek to engage with smokers at every opportunity, checking the smoking status of patients, advising those who smoke to quit and, where appropriate, making a referral to a stop smoking service 11. Primary care provides a key setting for such brief interventions and in 2004 the Quality and Outcomes Framework (QOF) was introduced which included incentivizing general practitioners to record patient smoking status and to offer cessation advice and/or an SSS referral 12. To our knowledge, no systematic review has yet been undertaken to explore SES differences in the delivery of such brief cessation interventions within primary care.

In recent years, there has been a marked change in patterns of SSS use and reach. The number of people setting a quit date with English services has fallen from approximately 725 000 (8.7% of all smokers) in 2012–13 to 275 000 (4.1% of smokers) in 2017–18 13. Similar declines in service use/reach have been shown in Scotland 14 and Northern Ireland 15, although the reverse trend has been seen in Wales, where the proportion of smokers making an SSS‐supported quit attempt increased from 1.1% in 2012–13 16 to 3.1% in 2017–18 17. SSS quit rates, in contrast, have remained broadly stable, with 4‐week abstinence rates of approximately 50–51% being reported, for example, in England 13. The net combined effect of these trends on SSS impact is unclear.

We provide here an updated and extended assessment of the contribution of UK stop smoking support to reducing socio‐economic inequalities in smoking. Building on the work of Brown *et al*. 8 and Bauld *et al*. 10, we combine a systematic review of the published literature with a separate national equity analysis of SSS reach and impact, also broadening the scope of our review to cover GP brief interventions as well as SSS. We address the following research questions:

1. How does (a) delivery of GP brief cessation interventions, (b) SSS use and quit rates and (c) SSS reach and impact, vary by smoker SES in the United Kingdom?
2. Which innovative stop smoking interventions demonstrate potential for improving cessation outcomes among low SES smokers?

## Methods

### Systematic review

**Figure 1:**
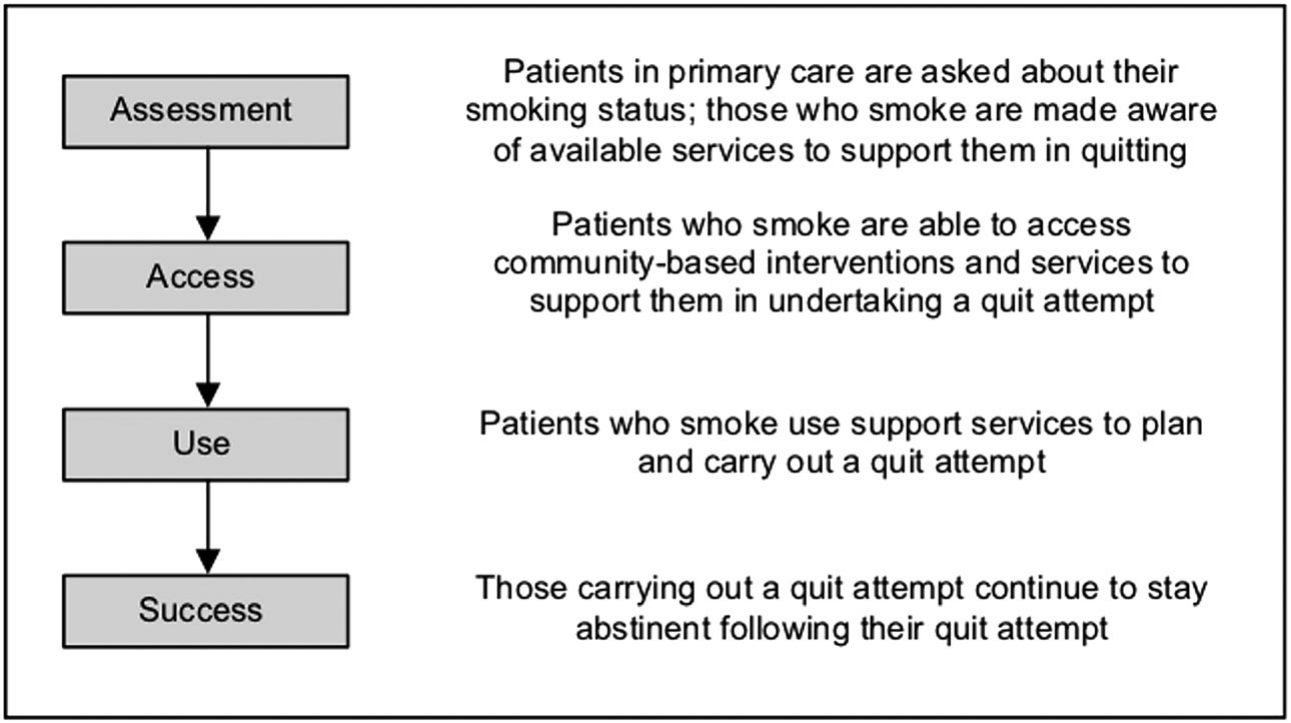
Conceptual model of the cessation pathway

This review was underpinned by a conceptual model of the cessation pathway describing the various steps involved in a successful quit attempt (Fig. 1), and was written according to the Preferred Reporting Items for Systematic Reviews and Meta‐Analyses (PRISMA): Equity Reporting Guidelines 18 (Supporting information, Appendix S1). The study protocol can be found at: http://doi.org/10.13140/RG.2.2.17572.17286.

### Eligibility criteria

Study eligibility criteria were: evaluated UK specialist or primary care‐based stop smoking support, published since 2012 in English, focused on adult participants (≥ 16 years) and reported at least one pathway‐related outcome (Table 1), and compared this outcome among two or more SES groups (or presented the findings for a specific disadvantaged group). The 2012 cut‐off was chosen for several reasons. Brown *et al*.'s systematic review 8 covered 13 UK SSS studies published between 2003 and 2012, predominantly reporting on data collected between 2000 and 2007. As no review of GP brief cessation interventions had previously been undertaken, a 2012 cut‐off gave a balance between minimizing overlap with the Brown review and including sufficient years to adequately capture latest evidence on GP brief interventions. Moreover, we intended to explore implications of the SSS transfer from National Health Service (NHS) to local authority control in 2013 19, and thus sought to include evidence gathered prior to this move. Ultimately, such analyses proved impossible, as the majority of eligible SSS studies were based on data collected between 2008 and 2013, with only three official statistics releases containing more recent information. Although two GP studies incorporated data back to the early 2000s, these studies also included longer‐term trend data to 2008–09, so we retained them in our analysis.

**Table 1:** Scope of systematic search.

Pathway‐related outcomes		Ascertainment of smoking status; receipt of brief cessation advice; engagement with services; quit attempts; use of behavioural support and/or pharmacotherapy; quit success
Bibliographic databases		Medline; Web of Science (Core Collection and BIOSIS); EMBASE; PsycINFO; ASSIA; CINAHL Plus; IBSS; Sociological Abstracts; Cochrane Library
Summary search terms	Block 1: Socio‐economic indicators	Education (including levels of literacy); income; occupational class; social grade; composite measures of individual disadvantage; area‐based measures of deprivation [including Carstairs, Townsend and Indices of Multiple Deprivation for England (IMD), Scotland (SIMD) and Northern Ireland (NIMDM)]; Mosaic consumer classification; prescription fee exemption, receipt of state benefits
	Block 2: Cessation interventions	GP brief interventions (including those delivered through NHS Health Check and QOF); stop smoking services (including behavioural support and pharmacotherapy); innovative GP and SSS‐based interventions (e.g. financial incentive schemes)
	Block 3: UK‐based	UK; Great Britain; England; Scotland; Wales; Northern Ireland
Research designs		Randomized controlled trials (RCTs); non‐randomized trials; cohort studies; cross−sectional surveys

### Search strategy and study selection

Ten electronic bibliographic databases (Table 1) were searched on 14 April 2017, using three blocks of search terms covering socio‐economic inequalities, smoking cessation and UK‐based research (Table 1 and Supporting information, Appendix S2A). Eligible papers had to appear in all three blocks, but there were no restrictions on the research designs employed. Relevant grey literature was sourced through key informants and online searches of SSS official statistics.

Duplicates were removed in Endnote by matching on combinations of four identifiers (title, author, year, journal) and manually reviewing possible matches. Titles and abstracts were screened by C.E.S. to identify those evaluating cessation interventions by SES or within a disadvantaged group. Selected articles were then subject to full‐text review to determine whether they met the study eligibility criteria. Results were independently checked by at least one other author, with any disagreements resolved through discussion.

### Data extraction and analysis

Standardized data extraction sheets were used to record the following for each eligible study: research design, location, years of data collection, sample characteristics, intervention type and setting, SES measures, cessation pathway steps and intervention outcomes by SES (Supporting information, Appendix S2B). Study quality was assessed using a modified version of the Critical Appraisal Skills Programme (CASP) tools 20, where checklists for randomized controlled trials (RCTs), cohort and case–control studies were combined into a single instrument (Supporting information Appendix S2C). Data extraction and quality appraisal was undertaken by C.E.S. Details were checked by A.A. or S.E.H., who also assessed each paper's overall value to the review (H: high; H/M: high/medium; M: medium; M/L: medium/low; L: low), taking into account both the individual CASP ratings and the relevance of the findings to our research questions. Quality appraisal results for each study are in Supporting information, Appendix S2D.

Wide variation in intervention types and outcomes meant that a meta‐analysis was not feasible. Findings are instead presented via narrative synthesis, with results reported separately by broad intervention type (GP, SSS, innovation) and pathway stage (assessment, access, use, success). Intervention equity impact was assessed for each SES indicator/outcome measure, using an adaptation of the criteria developed by Brown *et al*. 8 (Table 2). A similar rating system was used to classify the effectiveness of interventions targeted at disadvantaged groups. Equity impact ratings for each study finding can be found in Supporting information, Appendix S2E. Throughout the narrative synthesis, the overall value rating for the paper and the individual equity impact score for each finding (e.g. H,++) are included to indicate the strength and direction of evidence for that result.

**Table 2:** Equity impact classification criteria.

Positive [++]	Strong evidence that lower SES groups are relatively more responsive to intervention (either a robust measure from a national data set or supported by a formal statistical comparison showing a significant difference between groups) Example: NRT prescription in most versus least deprived: OR = 1.41, 95% CI = 1.11–1.80 (Blane *et al.* 23)
Possibly positive [+]	Some evidence that lower SES groups are relatively more responsive to intervention (either a weak measure from a national data set or a large difference between groups but study underpowered/no formal statistical analysis undertaken) Example: Smoking status recorded in most versus least deprived: pre‐QOF OR = 1.07, 95% CI = 0.93–1.23; post‐QOF OR = 1.35, 95% CI = 1.21–1.49 (Taggar *et al*. 12)
Neutral [o]	No evidence that intervention had differential impact across low and high SES groups (must be supported by a formal statistical analysis with reasonable power) Example: Quit success per unit increase in deprivation: OR = 0.98, 95% CI = 0.96–1.01 (Brose *et al*. 22)
Possibly negative [−]	Some evidence that higher SES groups are relatively more responsive to intervention (either a weak measure from a national data set or a large difference between groups but study underpowered/no formal statistical analysis undertaken) Example: GP cessation advice in most versus least deprived: OR = 0.80, 95% CI = 0.62–1.02 (Blane *et al*. 21)
Negative [– –]	Strong evidence that higher SES groups are relatively more responsive to intervention (either a robust measure from a national data set or supported by a formal statistical comparison showing a significant difference between groups) Example: Quit success in least versus most deprived: OR = 1.4, 95% CI = 1.1–1.9 (Bauld *et al*. 32)
Unclear [?]	Not able to assess intervention equity impact based on available evidence Example: Interaction between treatment type and SES non‐significant but OR/CIs not reported so unable to assess power. Wide CIs in main effect analysis of treatment type (Stapleton *et al*. 24)

OR = odds ratio; CI = confidence interval; NRT = nicotine replacement therapy; SES = socio‐economic status.

### National equity analysis: SSS reach and impact

Estimates of SSS reach and impact, broken down by SES, were calculated by combining data from several nationally representative reporting systems, including annual mid‐year population estimates, survey‐based estimates of smoking prevalence and SSS official statistics. Separate estimates were produced for each UK constituent nation (England, Scotland and Northern Ireland), but could not be generated for Wales as SES‐level data were unavailable. Comparison of each measure across the lowest and highest SES groups allowed an assessment of SSS equity impact by country. Sensitivity analyses explored the effect of missing SES data and coding inconsistencies on the robustness of the equity impact analysis. Full details (including data sources used) are in Supporting information, Appendix S2F.

## Results

### Systematic review

**Figure 2:**
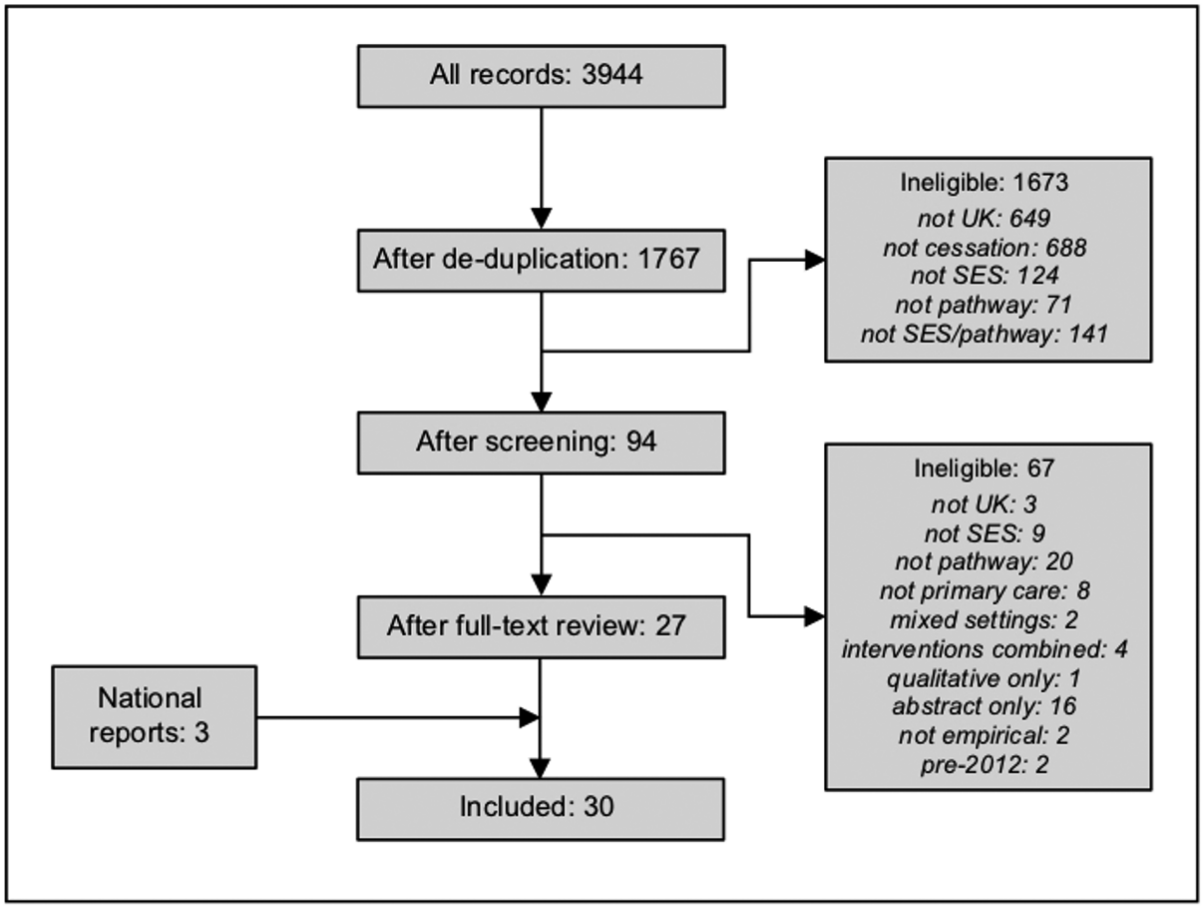
Preferred Reporting Items for Systematic Reviews and Meta‐Analyses (PRISMA) flow diagram

Bibliographic searches identified 3944 references which reduced to 1767 papers after removal of duplicates (Fig. 2). Initial screening of titles and abstracts led to 1673 articles being excluded, mainly because they did not involve UK data or did not focus on smoking cessation. Full‐text review of the remaining 94 papers identified 27 eligible articles.

Key informants pointed to seven possible further reports, but none contained data by SES. Online searches of SSS official statistics found 25 reports and workbooks. Two covered services in Wales, but neither presented findings by SES. The remaining 23 were collated to produce summary reports for England, Scotland and Northern Ireland, incorporating data for the time‐period 2009–17. Combined results from the academic and grey literature thus yielded 30 papers (27 academic articles; three national reports) for inclusion in the analysis. Table 3 provides brief study characteristics, the overall value rating and a summary equity impact assessment for each study (the full table of results is in Supporting information, Appendix S2E).

**Table 3:** Characteristics and summary findings of included studies.

	Study design	Population	Location	Years for SES analysis	Intervention type	Cessation‐related outcomes	SES measures^a^	Value to review^2^	Equity impact
GP brief interventions									
Blane *et al*. (2017) 21	Cross‐sectional	Smokers aged ≥ 25 with CHD	Scotland	2006–07	Routine care	Advice; prescription	Carstairs	H/M	Mixed
Dhalwani *et al*. (2013) 25	Before & after	Pregnant females aged 15–49	UK	2000–09	QOF	Smoking status	Townsend	H	Positive
Dhalwani *et al*. (2014) 26	Cross‐sectional	Pregnant smokers aged 15–49	UK	2006–12	Routine care	Prescription	Townsend	H	Positive
Douglas & Szatkowski (2013) 27	Cross‐sectional	Smokers aged ≥ 16	UK	2008–10	Routine care	Advice; prescription	Townsend; Mosaic	H/M	Positive
Forster *et al*. (2016) 28	Case–control	Healthy adults aged 40–74	England	2010–13	NHS Health Check	Smoking status	IMD	H	Possibly positive
Hamilton *et al*. (2016) 29	Before & after	Healthy adults aged ≥15	London	2006–11	QOF+	Smoking status; advice	IMD	H/M	Mixed
Hardy *et al*. (2014) 30	Cross‐sectional	Pregnant smokers aged 15–49	UK	2006–09	Routine care	Advice	Townsend	H/M	Positive
Taggar *et al*. (2012) 12	Before & after	Adults aged ≥ 15	UK	2002, 2004 & 2008	QOF	Smoking status; advice	Townsend	H/M	Possibly positive
Stop smoking services									
Bauld *et al*. (2012) 31	Cohort	Clients who set quit date	Liverpool & Knowsley	2009	Drop‐in rolling group	Success (52 weeks)	Composite	H	Negative
Bauld *et al*. (2016) 23	Cohort	Clients aged ≥ 16 who set quit date	England (9 areas)	2012–13	Mixed	Success (52 weeks)	Composite	H	Negative
Brose *et al*. (2012) 22	Cohort	Clients who make quit attempt	England	2009–11	One‐to‐one	Success (4 weeks)	Occupation; IMD; free prescription	H/M	Mixed
Brose *et al*. (2013) 32	Cohort	Clients who make quit attempt	England	2009–11	Mixed	Medication type; success (4 weeks)	Free prescription	H/M	Negative
Brose & McEwen (2016) 33	Cohort	Clients who make quit attempt	England	2009–12	Mixed	Compliance; medication type; success (4 weeks)	IMD; free prescription	H	Negative
DoH NI (2016) 34	Cohort	Clients who make quit attempt	Northern Ireland	2009–16	Mixed	Attempts; success (4 weeks)	NIMDM	H	Mixed
Hiscock *et al*. (2013) 35	Cohort	Clients who make quit attempt	England	2010–11	Mixed	Support type; success (4 weeks)	Occupation; free prescription	H/M	Mixed
Hiscock *et al*. (2015) 36	Cohort	Clients aged ≥16 who set quit date	England (9 areas)	2012–13	Mixed	Support type; success (52 weeks)	Composite	H/M	Mixed
ISD Scotland (2017) 37	Cohort	Clients who make quit attempt	Scotland	2009–17	Mixed	Attempts; success (4 & 12 weeks)	SIMD	H	Mixed
McAlpine *et al*. (2015) 38	Cohort	Clients aged ≥ 18 who set quit date	London	2013–14	Mixed	Success (4 weeks)	Occupation	L	Unclear
NHS Digital (2017) 39	Cohort	Clients who make quit attempt	England	2009–17	Mixed	Attempts; success (4 weeks)	Occupation	H	Mixed
West *et al*. (2013) 40	Cross‐sectional	Clients who set quit date	England	2008–11	Mixed	Attempts	Free prescription	M/L	Possibly positive
Innovations									
Bennett *et al*. (2015) 41	RCT	Smokers aged 18–65 registered with GP	UK	2007–08	GP communications	Success (3 months)	Literacy level	M	Possibly positive
Gilbert *et al*. (2017) 42	RCT	Smokers aged ≥ 16 registered with GP	England	2011–14	GP communications	Uptake; success (6 months)	IMD	M	Possibly negative
Maskrey *et al*. (2015) 43	RCT	SSS clients who abstinent at 4 weeks	East of England	2011–13	SSS relapse prevention booklet	Abstinence (12 months)	Education; free prescription	H/M	Unclear
Stapleton *et al*. (2013) 24	RCT	SSS clients	South East England	2004–07	Pharmacotherapy	Success (6 months)	Education; state benefits	L	Unclear
Turner *et al*. (2013) 44	Cohort	SSS clients who abstinent at 8 weeks	Nottingham	2010–11	NRT for relapse prevention	Accept extended course of NRT	Occupation; free prescription	M/L	Mixed
Venn *et al*. (2016) 45	Non‐randomized	Smokers who live or work in area	Nottingham	2011	Mobile SSS versus one‐to‐one SSS	Uptake; success (4 weeks)	Multiple	M	Mixed
Kassim *et al*. (2016) 46	Cohort	Healthy ethnic minority smokers aged ≥18	London	2007–08	Community outreach SSS	Success (4 weeks)	Deprived area	M	Possibly positive
Ormston *et al*. (2015) 47	Quasi‐experiment	Smokers from deprived areas	Dundee	2007–11	Incentives (mixed settings)	Attempts; success (1, 3 & 12 months)	Deprived area	H/M	Mixed
Radley *et al*. (2013) 48	Cohort	Pregnant smokers	Tayside	2007–09	Incentives (pharmacy)	Attempts; success (4 & 12 weeks)	Deprived area	M/L	Positive
Tappin *et al*. (2015) 49	RCT	Pregnant smokers aged ≥16	Glasgow	2011–13	Incentives (SSS)	Attempts; success (8 weeks & 6 months)	Deprived area	H/M	Mixed

a Details of the SES measures used can be found in Table 1.

2 Value to review rating—H: high; H/M: high/medium; M: medium; M/L: medium/low; L: low. CHD = coronary heart disease; GP = general practitioner; SES = socio‐economic status; RCT = randomized controlled trial; IMD = index of multiple deprivation; DoH NI = Department of Health Northern Ireland; ISD = Information Services Division; QOF = Quality and Outcomes; SSS = Stop Smoking Secvices.

### Characteristics of included studies

Two‐thirds of included studies investigated the equity impact of existing stop smoking support, with eight assessing delivery of GP brief interventions (through both routine care and specific initiatives such as the NHS Health Check) and 12 focusing on SSS. The remaining 10 evaluated innovative interventions in diverse settings. A wide variety of outcome measures were considered, covering all stages of the cessation pathway: assessment (recording smoking status), access (receiving cessation advice and engaging with interventions), use (quit attempts and support usage) and success (quit rates).

### Delivery of GP brief interventions (eight studies)

**Figure 3:**
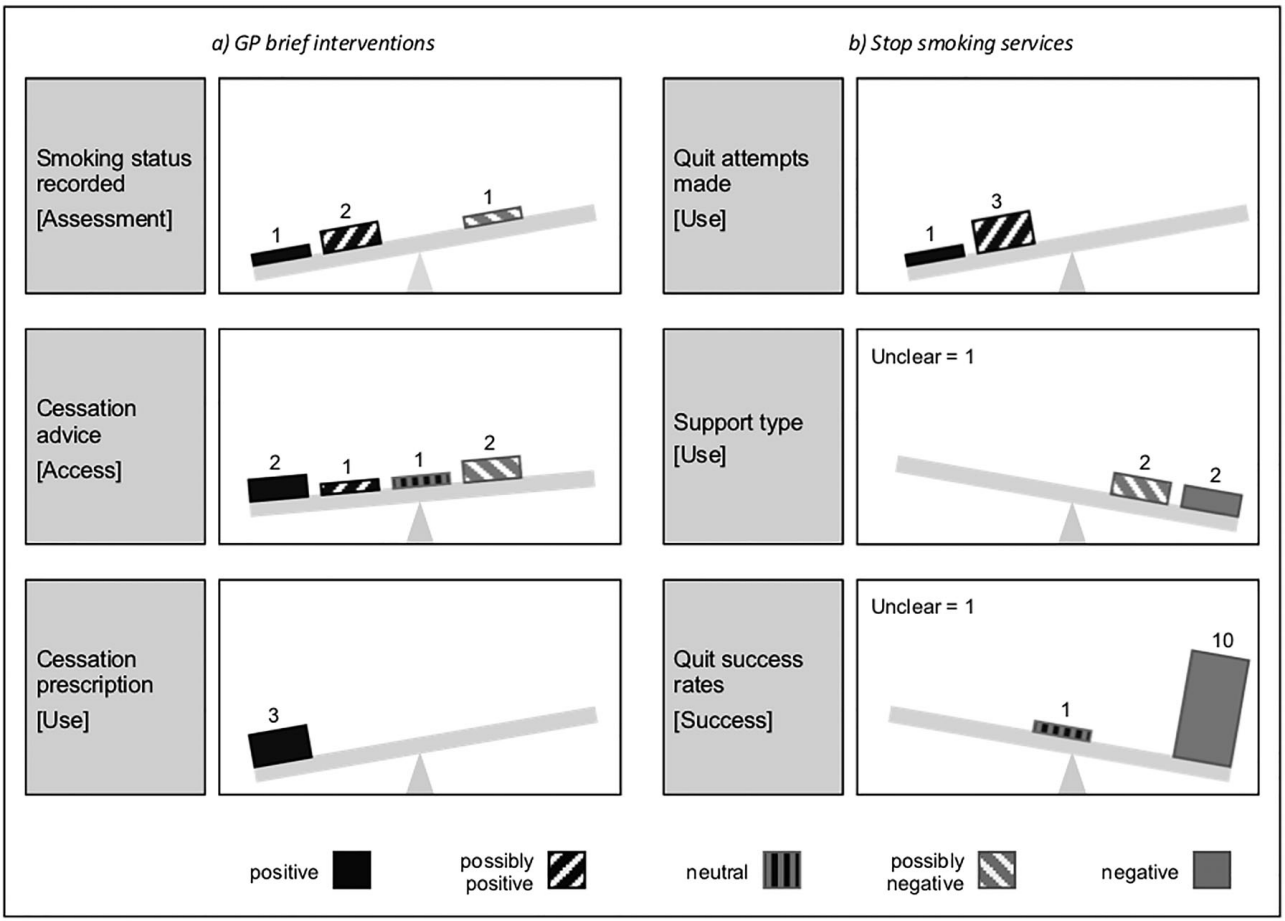
Equity impact of existing stop smoking support

Four papers assessed the recording of smoking status in patients’ GP notes (Fig. 3a), with three evaluating the impact of the Quality and Outcomes Framework (QOF), a scheme which incentivized GPs to record smoking status and give brief cessation advice (among other performance indicators) 12. UK‐wide studies by Dhalwani *et al*. 25 (H,++) and Taggar *et al*. 12 (H/M,+) found that, after introduction of QOF, the greatest improvement in recording of smoking status was seen for residents of the most disadvantaged areas. In contrast, an enhanced local version of the scheme (QOF+) in one London borough had a negative equity impact (H/M,–) 29. Forster *et al*. 28 meanwhile examined the effect of the NHS Health Check (a national preventive programme to reduce cardiovascular morbidity) in England, showing that SES inequalities in recording smoking status were no longer apparent after the programme was implemented (H,+).

Of the five papers examining provision of brief cessation advice (including SSS referral), two national studies [Douglas & Swatkowski 27 (H/M,++); Taggar *et al*. 12 (H/M,+)] showed that advice was more commonly recorded in notes for smokers from the most deprived areas of the United Kingdom. Some variation was apparent across patient subgroups: at a UK‐level, Hardy *et al*. 30 (H/M,++) found brief cessation advice during pregnancy was more likely to be given to low SES smokers; while in Scotland, Blane *et al*. 21 (H/M,–) showed that patients diagnosed with coronary heart disease (CHD) were less likely to receive cessation advice if they lived in disadvantaged areas. The London‐based enhanced QOF+ (H/M) 29 had a neutral equity impact in males but a negative impact in females.

Finally, three GP studies investigated prescriptions for cessation medication. Here, disadvantaged smokers were more likely to receive a prescription for nicotine replacement therapy both among pregnant patients in the United Kingdom 26 (H,++) and among patients with CHD in Scotland 21 (H/M,++). More broadly, low SES smokers in the United Kingdom were more likely to be prescribed cessation medication of any kind 27 (H/M,++).

### SSS use and quit rates (12 studies)

Evidence relating to quit attempts (service use) came primarily from the collated official statistics, with all three reports showing that services in England 39 (H,+), Scotland 37 (H,++) and Northern Ireland 34 (H,+) succeeded in attracting more low than high SES smokers (Fig. 3b). A separate analysis of service monitoring data for English SSSs 40 (M/L,+) similarly reported an equity‐positive effect.

Four papers investigated variations in use of pharmacotherapy and behavioural support. Several studies showed that varenicline was less commonly prescribed for low SES smokers 32, 33, 36 (H/M,– –; H,– –;H/M,–). Findings were more equivocal with respect to behavioural support: one study 36 (H/M,–) found that disadvantaged clients were more likely to use the less effective drop‐in services, but another 35 (/M,?) demonstrated similar patterns of engagement throughout SES groups (although this latter finding was not subject to formal statistical testing).

The most frequently evaluated outcome measure was quit success (service quit rates), with 11 papers assessing socio‐economic differences in quit rates among SSS clients. In addition to the three national reports 34, 37, 39 (H (3)], six studies reported data for England based on a sample of services 22, 23, 32, 33, 35, 36 [H (2); H/M (4)]. Almost all these analyses indicated a negative equity impact over a range of time‐points (from 4 to 52 weeks) and SES indicators. The one exception was Brose *et al*. 22 (H/M,o/− −), who found that 4‐week quit rates for one‐to‐one support did not vary by the index of multiple deprivation 50 (IMD), although a negative equity effect was apparent by occupational status. Two further studies focused on single services within deprived areas, one reporting falling quit rates with increasing disadvantage for users of drop‐in rolling support groups in Liverpool 31 (H,– –), and the other presenting descriptive data only for a SSS in London 38 (L,?).

### Innovative stop smoking interventions (10 studies)

Studies of innovative interventions were divided between those comparing outcomes across SES groups and those focusing solely on disadvantaged smokers (Table 4). Of the six studies assessing equity impact, two investigated interventions tailored towards low SES smokers. Bennett *et al*. 41 (M,+) conducted an RCT of GP‐endorsed cessation advice where computer‐tailored reports were matched to the smoker's literacy level. The intervention effect on quit rates was greater for the easy‐reading than the standard‐reading group, although wide confidence limits meant differences were not statistically significant. Venn *et al*. 45 (M) evaluated a mobile stop smoking service (MSSS), which toured deprived areas of Nottingham. This gave mixed results in relation to access, with an equity‐positive effect (++) in terms of the proportion of service users from manual occupations compared to the standard SSS for the same area, but no equity impact (o) for two further SES measures (area deprivation and prescription fee exemption). Among MSSS users, quit rates were also higher (+) for manual workers than for all clients, although quit rates across all client groups were worse compared to the standard service. Four comparative studies explored non‐tailored interventions, with only one 44 (M/L) finding limited evidence of an equity‐positive effect: SSS clients from manual occupations were more likely to use nicotine replacement therapy (NRT) for relapse prevention than those from professional groups (+) or the unemployed (– –).

**Table 4:** Details of innovative interventions.

Studies of equity impact	
Bennett *et al.* (2015) 41	Customized cessation advice matched to smoker's reading level plus brief endorsement letter from GP (SES tailored)
Gilbert *et al*. (2017) 42	Customized risk letter from GP plus invitation to attend a no‐commitment taster session at a local stop smoking service
Maskrey *et al*. (2015) 43	Pack of relapse prevention booklets distributed through stop which aimed to help quitters recognize high‐risk relapse situations and give them the skills to cope in such situations
Stapleton *et al*. (2013) 24	RCT of three forms of pharmacotherapy (NRT alone, bupropion alone, and combination NRT + bupropion) delivered through stop smoking services (SSS)
Turner *et al*. (2013) 44	Extended course of NRT for relapse prevention given to SSS clients who remained successfully quit at 4 weeks
Venn *et al*. (2016) 45	Mobile drop‐in, community‐based stop smoking service which sought to improve reach among disadvantaged smokers (SES tailored)
Studies of interventions targeted at disadvantaged groups	
Kassim *et al*. (2016) 46	Community‐based outreach stop smoking service with opportunity to receive support in smoker's native language from adviser of same gender
Ormston *et al*. (2015) 47	Financial incentives scheme (quit4u) targeted at smokers living in deprived areas delivered across a range of primary care settings
Radley *et al*. (2013) 48	Financial incentives scheme (Give It Up for Baby) targeted at pregnant smokers in deprived areas delivered through community pharmacies
Tappin *et al*. (2015) 49	Financial incentives scheme targeted at pregnant smokers in deprived areas delivered through community‐based stop smoking services

GP = general practitioner; RCT = randomized controlled trial; NRT = nicotine replacement therapy; SES = socio‐economic status.

The remaining four papers assessed the efficacy of innovations targeting smokers from disadvantaged areas. Three explored the impact of financial incentive schemes in Scotland: a cohort study of a pharmacy‐based intervention for pregnant smokers 48 (M/L), an RCT of an SSS‐administered scheme for pregnant smokers 49 (H/M) and a quasi‐experimental evaluation of a programme for all residents delivered across various primary care settings 47 (H/M). Only Radley *et al*. 48 found an improvement in quit attempts, but all three schemes demonstrated higher quit success rates than those reported across a range of other comparator interventions. The final targeted innovation 46 (M,+) was a community outreach SSS for smokers from a minority ethnic group (British Bangladeshis), offering one‐to‐one cessation support to clients in their native language from gender‐matched community workers. This reported better quit outcomes than the standard SSS.

### National equity analysis: SSS reach and impact

**Figure 4:**
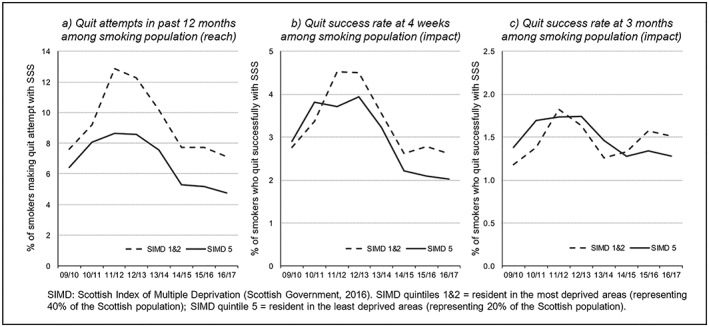
Reach and impact of stop smoking services (SSSs) in Scotland by socio‐economic status (SES), 2009–17

Robust estimates of SSS reach and impact by SES were only available for Scotland. Here, smokers living in the most deprived areas (SIMD1 and 2) were more likely to attempt to quit with SSS support than those in the least disadvantaged areas (SIMD5), with an equity‐positive effect on reach being evident throughout the period 2009–17 (Fig. 4a). SSS impact at 4 weeks (Fig. 4b) was initially higher for the most affluent smokers, but this pattern reversed from 2011 onwards, following the introduction of deprivation‐based equity targets for Scottish SSSs (HEAT targets) 51. In contrast, 3‐month SSS impact remained equity‐negative (apart from briefly during 2011) until further changes to the SSS equity targets in 2014 (shifting the focus to more sustained quitting) saw the rate for disadvantaged smokers overtake that for affluent smokers (Fig. 4c). These results suggest that SSS in Scotland may have an equity‐positive effect on smoking inequalities, with successive refinements of the equity targets possibly playing a role.

Equivalent SSS reach and impact analyses for English SSSs were inconclusive, due to issues with availability and consistency of SES measures among source data sets. Results in Northern Ireland were similarly affected by missing SES data, although recent improvements in recording meant that more reliable estimates could be produced for 2015–16. Here, comparison of the most and least disadvantaged areas suggests that SSS in Northern Ireland may also have an equity‐positive effect on service reach and impact. Full details of the national equity/sensitivity analyses for England and Northern Ireland are in Supporting information, Appendix S2(F).

## Discussion

Our review, covering 27 academic articles and three national reports, suggests that stop smoking support in the United Kingdom plays an important role in addressing socio‐economic inequalities in smoking through a combination of reach and support. GP brief interventions were equity‐positive in terms of identifying disadvantaged smokers and encouraging them to quit: low SES groups were more likely to have their smoking status recorded, be offered brief cessation advice and/or SSS referral and be provided with cessation medication. SSS attracted higher numbers of disadvantaged smokers, although results were less positive in relation to quit rates which were consistently lower among deprived client groups. There was also some evidence that low SES smokers were less likely to be prescribed varenicline, the most effective form of pharmacotherapy 52. While evidence was more limited regarding SSS reach and impact, equity results were nevertheless encouraging. Increased SSS reach among low SES smokers in Scotland, and possibly also Northern Ireland, more than compensated for lower quit rates, resulting in an overall equity‐positive effect on impact. Several innovative approaches showed promise, including interventions that could be offered through existing channels (e.g. incentive schemes for smokers 47, 48, 49, tailored advice matched to literacy levels 41) and interventions targeted at disadvantaged groups (e.g. mobile 45 or outreach 46 services in low SES communities).

Ours is the first systematic review, to our knowledge, to examine socio‐economic differences in the delivery of brief cessation interventions within primary care, and we demonstrate that GPs in the United Kingdom have been particularly successful at engaging and supporting low SES smokers. The QOF 12 was introduced in 2004 with the aim of incentivizing GPs to deliver such brief interventions, and our review suggests that QOF may have been especially effective at improving intervention rates in disadvantaged smokers, potentially helping to reduce inequalities in cessation and smoking. These advances are, however, under threat following the discontinuation of QOF in Scotland from 2016, and amid growing dissatisfaction with the framework in England 53. Alongside this, there have been significant recent declines in primary care prescriptions for smoking cessation medications throughout the United Kingdom, with some parts of England issuing guidance to GPs discouraging the prescription of such medications 54, possibly endangering the equity‐positive pattern of prescribing identified in the current review. A lack of evidence gathered since 2013 limits our ability to assess the effects of these policy developments, emphasizing the need for continued research investment.

In line with past studies 7, 8, 9, we show that quit success rates among disadvantaged SSS clients continue to lag behind those of their more affluent counterparts. Unlike the review by Brown *et al*. 8, we found no studies that examined equity effects in relation to SSS reach and impact, although we were able to produce such an analysis for Scotland. Despite steep declines in recent years in the numbers making an SSS‐supported quit attempt 14, we provide evidence that Scottish services have maintained a net equity‐positive effect on quit success rates in the smoking population. Our results also suggest a possible role for equity‐based SSS performance targets of the type introduced by the Scottish Government 51. The rapid drop in SSS use is apparent across the United Kingdom and, while specific reasons for this remain unclear, contributing factors may include falling investment in services 19, 55, the transfer of English SSS to local authority control (with some smokers no longer having access to a stop smoking service) 19, reduced use of mass media campaigns 55 and the emergence of e‐cigarettes as a cessation aid 56. Given the lack of recent data (particularly for England and Wales), and in light of changes in the organization, funding and use of SSS, research is required to determine whether services in all parts of the United Kingdom have been able to sustain their previous equity‐positive effect 8, 10 at the population level.

This systematic review was underpinned by a conceptual model of the cessation pathway and was comprehensive and inclusive in scope, encompassing a wide range of research designs, socio‐economic indicators and outcome measures. This approach enabled us to consider stop smoking support at every stage of the cessation pathway (including GP attempts to engage with smokers and encourage them to quit), and not just during the actual quit itself. Nevertheless, our study has several limitations. First, in keeping with previous reviews 8, 9, only a third of the identified studies were designed with the primary aim of evaluating equity impact, although several papers focused on the experiences of disadvantaged smokers. Thus, while the quality of many of the studies was high in relation to their main research question, we encountered problems (e.g. low statistical power and a lack of adequate comparators) when trying to assess socio‐economic differences in cessation support. Moreover, while we attempted to source relevant grey literature in order to reduce the potential impact of publication bias (the tendency of academic journals to publish research that demonstrates an intervention effect) 57, none of the reports identified by key informants contained SES data. It is possible that equity‐neutral findings are under‐represented within our review. Next, as we were unable to conduct a meta‐analysis due to wide variation in intervention types and outcome measures, we presented instead a narrative synthesis accompanied by a visual representation of the equity impact findings using a vote‐counting approach (Fig. 3). Here, we sought to mitigate difficulties with the interpretation of borderline or non‐statistically significant results 57 by using possibly positive‐/negative‐equity impact ratings for studies that were underpowered or that contained no formal statistical analysis. Nevertheless, caution in interpretation is still required, as this graphical approach assumes equal weighting of all study findings 57. Finally, our search identified only one qualitative paper 58 (exploring disadvantaged smokers’ perceptions of the mobile stop smoking service described by Venn *et al*. 45) which could not be usefully integrated into our analysis. This dearth of qualitative evidence somewhat restricts our understanding of the reasons why certain interventions might be more successful in achieving an equity‐positive effect than others.

In conclusion, this review highlights the potential of stop smoking support in helping to reduce socio‐economic inequalities in smoking. Primary care providers and stop smoking services can together provide support to smokers across all stages of a quit attempt, facilitating a ‘joined‐up’ approach to cessation that may be particularly important for those from lower socio‐economic groups. High levels of SSS uptake among low SES smokers are essential to compensate for their lower rates of quit success, and brief interventions by GPs that identify and channel disadvantaged smokers towards appropriate forms of behavioural support and pharmacotherapy are key to accomplishing this. Changing models of funding and service delivery can threaten the success of such support, emphasizing the need for sustained commitment and investment in the development and delivery of cessation support targeted at disadvantaged smokers.

## Declaration of interests

None.
