# Supplementary material for: Impact of specialist and primary care stop smoking support on socio‐economic inequalities in cessation in the United Kingdom: a systematic review and national equity initial review completed 22 January 2019; final version accepted 19 July 2019 analysis
Source: Addiction. 2019 Sep 10;115(1):34–46. doi: 10.1111/add.14760 (PMC6973008; doi:10.1111/add.14760)
Supplement: Supplementary file 2 [file ADD-115-34-s002.docx]

**Impact of specialist and primary care stop smoking support on socioeconomic inequalities in cessation in the UK: a systematic review and national equity analysis**

Caroline Smith, Amanda Amos, Sarah Hill

*Appendix S2*

**CONTENTS PAGE**

1. Search terms applied in MEDLINE 2-3
2. Data extraction sheet 4
3. Quality appraisal checklist & comparison with CASP instruments 5-6
4. Quality appraisal ratings 7
5. Equity impact & intervention effectiveness ratings 8-11
6. National equity impact estimates: methods & sensitivity analyses 12-13
7. **Search terms applied in MEDLINE**

| *Search no.* | *Search terms* |
| --- | --- |
| Search block: socioeconomic status | |
| 1 | exp Socioeconomic Factors/ |
| 2 | exp Public Assistance/ |
| 3 | exp Social Welfare/ |
| 4 | Vulnerable Populations/ |
| 5 | (socioeconomic or socio economic or socio-economic).ti,ab. |
| 6 | SES.ti,ab. |
| 7 | (social adj (class$ or group$ or grade$ or context$ or status)).ti,ab. |
| 8 | (social$ adj (disadvant$ or exclusion or excluded or depriv$)).ti,ab. |
| 9 | (townsend or carstairs or mosaic or health action zone$ or priority group$ or "index of multiple deprivation" or IMD or SIMD or WIMD or NIMDM).ti,ab. |
| 10 | demographic$.ti,ab. |
| 11 | (inequal$ or equity).ti,ab. |
| 12 | (depriv$ or disadvantage$ or poverty or poor).ti,ab. |
| 13 | (uninsur$ or insur$).ti,ab. |
| 14 | affluen$.ti,ab. |
| 15 | (underserved or under served or under-served).ti,ab. |
| 16 | (high risk or high-risk or at risk).ti,ab. |
| 17 | (marginalised or marginalized).ti,ab. |
| 18 | (employ$ or unemploy$).ti,ab. |
| 19 | occupation$.ti,ab. |
| 20 | (work site or worksite or work-site).ti,ab. |
| 21 | (work place or workplace or work-place).ti,ab. |
| 22 | (work force or workforce or work-force).ti,ab. |
| 23 | income.ti,ab. |
| 24 | educat$.ti,ab. |
| 25 | minorit$.ti,ab. |
| 26 | (area or region or geographic$ or north-south or north south).ti,ab. |
| 27 | 1 or 2 or 3 or 4 or 5 or 6 or 7 or 8 or 9 or 10 or 11 or 12 or 13 or 14 or 15 or 16 or 17 or 18 or 19 or 20 or 21 or 22 or 23 or 24 or 25 or 26 |
| Search block: smoking cessation | |
| 28 | Smoking Cessation/ |
| 29 | "Tobacco Use Cessation"/ |
| 30 | "Tobacco Use Cessation Products"/ |
| 31 | Smoking/dt, pc, th [Drug Therapy, Prevention & Control, Therapy] |
| 32 | smoking cessation.kw. |
| 33 | (smoking and cessation).ti,ab. |
| 34 | cessation support.ti,ab. |
| 35 | ((stop$ or quit$ or ceas$ or reduc$ or giv$ up) adj3 (cigarette$ or tobacco or nicotine or smok$)).ti,ab. |
| 36 | ((cigarette$ or tobacco or nicotine or smok$) adj3 (cessation or abstinen$)).ti,ab. |
| 37 | ((quit$ or cessation) adj (attempt$ or outcome$ or rate$ or relaps$ or maintain$)).ti,ab. |
| 38 | (quitline$ or quit line$ or quit-line$).ti,ab. |
| 39 | ((smok$ or quit$ or tobacco) adj (helpline$ or help line$ or help-line$)).ti,ab. |
| 40 | (smokefree or smoke-free or smoke free).ti,ab. |
| 41 | 28 or 29 or 30 or 31 or 32 or 33 or 34 or 35 or 36 or 37 or 38 or 39 or 40 |
| Search block: general health promotion initiatives | |
| 42 | ((health or wellbeing or well-being or well being) adj (check$ or assess$)).ti,ab. |
| 43 | (NHS choices or NHS 24 or live well or keep well or "add to your life" or one you).ti,ab. |
| 44 | (healthy living adj (pharma$ or champion$)).ti,ab. |
| 45 | ("quality and outcomes framework" or QOF).ti,ab. |
| 46 | 42 or 43 or 44 or 45 |
| 47 | 41 or 46 |
| 48 | 27 and 47 |
| Search block: geographical location | |
| 49 | (UK or "U.K." or United Kingdom or Great Britain or Britain or British).ti,ab. |
| 50 | (England or English).ti,ab. |
| 51 | (Scotland or Scottish).ti,ab. |
| 52 | (Wales or Welsh).ti,ab. |
| 53 | (Northern Ireland or NI or "N.I." or Northern Irish).ti,ab. |
| 54 | 49 or 50 or 51 or 52 or 53 |
| 55 | 48 and 54 |
| 56 | limit 59 to (abstracts and english language and yr="2012 -Current") |

1. **Data extraction sheet**

| Study identifiers & initial descriptors | | | | |
| --- | --- | --- | --- | --- |
| Authors: |  | |  |  |
| Title: |  | |  |  |
| Journal: |  | |  |  |
|  | | | | |
| Study design: |  | | | |
| Location: |  | | | |
| Year(s) of data collection: |  | | | |
| Sample characteristics: |  | | | |
| SES variable: |  | | | |
| Intervention type & setting: |  | | | |
| Cessation pathway: |  | | | |
| Findings by SES group: |  | | | |
| Quality assessment for SES appraisal | | | | |
| *Checklist item* | *Code* | *Comments* | | |
| SES/cessation focus: |  |  | | |
| Data for SES appraisal: |  |  | | |
| SES indicator: |  |  | | |
| Confounding addressed: |  |  | | |
| Endpoint measurement: |  |  | | |
| Response rates: |  |  | | |
| Adequate power: |  |  | | |
| Generalisability: |  |  | | |
| Value for review: |  |  | | |

1. **Quality appraisal checklist & comparison with CASP instruments**

| *Checklist items* | *Associated CASP items^(i)^* |
| --- | --- |
| Did the paper focus primarily on SES experiences of smoking cessation interventions? [Yes/Partial/No] | R1. Did the trial address a clearly focused issue? |
|  | C1. Did the study address a clearly focused issue? |
|  | CC1. Did the study address a clearly focused issue? |
| Does the study include data that allow us to look at SES experiences of smoking cessation interventions? [Yes/Mixed/No/Unclear] | CC2. Did the authors use an appropriate method to answer their question |
| Were the SES indicators used adequate? [Yes/Mixed/No/Unclear] | C3. Was the exposure accurately measured to minimise bias? |
| Did the study design &/or analysis seek to minimise bias in group comparisons (intervention &/or SES) by taking account of potential confounding factors? [Yes/Partial/Mixed/No/Unclear/Not applicable] | R2. Was the assignment of patients to treatments randomised? |
|  | R4. Were patients, health workers and study personnel ‘blind’ to treatment? |
|  | R5. Were the groups similar at the start of the trial? |
|  | R6. Aside from the experimental intervention, were the groups treated equally? |
|  | C2. Was the cohort recruited in an acceptable way? |
|  | CC3. Were the cases recruited in an acceptable way? |
|  | CC4. Were the controls selected in an acceptable way? |
|  | C5. Have the authors identified all important confounding factors? |
|  | C6. Have they taken account of the confounding factors in the design and/or analysis? |
|  | CC6. What confounding factors have the authors accounted for? |
|  | CC7. Have the authors taken account of the potential confounding factors in the design and/or in their analysis? |
|  | CC5. Was the exposure accurately measured to minimise bias? |
| Were the relevant endpoints^(ii)^ accurately measured to minimise bias? [Yes/Mixed/No/Unclear] | C4. Was the outcome accurately measured to minimise bias? |
|  | R10. Were all clinically important outcomes considered? |
| Were response rates (initial &/or follow-up surveys) sufficient across interventions &/or SES groups to allow for unbiased & valid conclusions to be drawn? [Yes/Mixed/No/Unclear/Not applicable] | R3. Were all of the patients who entered the trial properly accounted for at its conclusion? |
|  | C7. Was the follow up of subjects complete enough? |
|  | C8. Was the follow up of subjects long enough? |
| Is the study likely to be adequately powered for the SES comparisons? [Yes/Mixed/No/Unclear/Not applicable] | R7. How large was the treatment effect? |
|  | R8. How precise was the estimate of the treatment effect? |
|  | C10. How precise are the results? |
|  | C11. Do you believe the results? |
|  | CC9. How precise are the results? |
|  | CC10. How precise is the estimate of risk? |
|  | CC11. Do you believe the results? |
| Can the study findings in relation to SES experiences of smoking cessation interventions be generalised and, if so, at what level? [National/Local/Mixed/No/Unclear] | R9. Can the results be applied in your context? (or to the local population?) |
|  | C12. Can the results be applied to the local population? |
|  | CC12. Can the results be applied to the local population? |
| Comment on value of study for our review (cessation experience by SES) | QL10. How valuable is the research? |
|  |  |
| Items excluded as relate more to description, practical issues, contextualisation & interpretation than to quality assessment | CC8. What are the results of this study? |
|  | C9. What are the results of this study? |
|  | R11. Are the benefits worth the harms and costs? |
|  | C13. Do the results of this study fit with other available evidence? |
|  | CC13. Do the results of this study fit with other available evidence? |
|  | C14. What are the implications of this study for practice? |

(i) R: checklist for RCTs; C: checklist for cohort studies; CC: checklist for case-control studies

(ii) Relevant endpoints can relate to any point in the cessation pathway, including assessment, access, use & success

1. **Quality appraisal ratings^(i)^**

|  | SES/cessation focus | Data for SES appraisal | SES indicator | Confounding addressed | Endpoint measurement | Response rates | Adequate power | Generalisable | Value to review |
| --- | --- | --- | --- | --- | --- | --- | --- | --- | --- |
| Blane et al (2017)^21^ | N | Y | Y | Y | M | NA | M | Y | H/M |
| Dhalwani et al (2013)^25^ | P | Y | Y | Y | U | NA | Y | Y | H |
| Dhalwani et al (2014)^26^ | P | Y | Y | P | Y | NA | Y | Y | H |
| Douglas & Szatkowski (2013)^27^ | Y | Y | Y | P | U | NA | Y | Y | H/M |
| Forster et al (2016)^28^ | P | Y | Y | Y | Y | NA | Y | Y | H |
| Hamilton et al (2016)^29^ | Y | Y | Y | Y | Y | NA | M | S | H/M |
| Hardy et al (2014)^30^ | P | Y | Y | Y | U | NA | Y | Y | H/M |
| Taggar et al (2012)^12^ | P | Y | Y | Y | U | NA | Y | Y | H/M |
| Bauld et al (2012)^31^ | Y | Y | Y | Y | Y | Y | Y | U | H |
| Bauld et al (2016)^23^ | N | Y | Y | Y | Y | Y | Y | U | H |
| Brose et al (2012)^22^ | N | Y | M | Y | Y | U | Y | U | H/M |
| Brose et al (2013)^32^ | N | Y | N | P | Y | U | Y | Y | H/M |
| Brose & McEwen (2016)^33^ | Y | Y | Y | Y | Y | U | Y | Y | H |
| DoH NI (2016)^34^ | - | - | - | - | - | - | - | - | H^(ii)^ |
| Hiscock et al (2013)^35^ | Y | Y | M | M | M | U | U | U | H/M |
| Hiscock et al (2015)^36^ | Y | Y | Y | Y | Y | Y | U | U | H/M |
| ISD Scotland (2017)^37^ | - | - | - | - | - | - | - | - | H^(ii)^ |
| McAlpine at al (2015)^38^ | Y | Y | Y | N | U | U | NC | N | L |
| NHS Digital (2017)^39^ | - | - | - | - | - | - | - | - | H^(ii)^ |
| West et al (2013)^40^ | Y | N | N | N | U | NA | U | Y | M/L |
| Bennett et al (2015)^41^ | Y | Y | U | Y | U | U | N | Y | M |
| Gilbert et al (2017)^42^ | N | Y | Y | U | Y | N | N | N | M |
| Maskrey et al (2015)^43^ | N | Y | M | Y | Y | U | Y | S | H/M |
| Stapleton et al (2013)^24^ | P | Y | M | Y | Y | U | NC | U | L |
| Turner et al (2013)^44^ | N | M | M | N | Y | NA | N | U | M/L |
| Venn et al (2016)^45^ | Y | Y | M | N | M | N | M | U | M |
| Kassim et al (2016)^46^ | Y | Y | Y | N | Y | U | U | S | M |
| Ormston et al (2015)^47^ | Y | Y | Y | M | M | M | Y | U | H/M |
| Radley et al (2013)^48^ | Y | Y | Y | N | U | N | M | N | M/L |
| Tappin et al (2015)^49^ | Y | Y | Y | Y | Y | N | Y | S | H/M |

1. M: mixed; NC: not possible to draw any conclusions; P: partial; S: generalisable within subpopulation; U: unclear
2. Formal quality appraisal of the SSS annual reports was not undertaken. As these publications are classed as Official Statistics releases and as they represent the sole source of national data covering all SSS clients, they were rated as high value for the purposes of this review.
3. **Equity impact & intervention effectiveness ratings**

*GP brief interventions*

| Study | Intervention | Outcome measure | SES measure | Equity impact | Findings |
| --- | --- | --- | --- | --- | --- |
| Assessment | | | | | |
| Dhalwani et al (2013)^25^ | QOF | Smoking status recorded | Townsend | Positive  [H,++] | Adj. OR for most vs least deprived:  pre-QOF = 1·17 (1·10-1·25)  post-QOF = 1·42 (1·37-1·47) |
| Taggar et al (2012)^12^ | QOF | Smoking status recorded | Townsend | Possibly positive  [H/M,+] | Adj. OR for most vs least deprived:  pre-QOF = 1·07 (0·93-1·23)  post-QOF = 1·35 (1·21-1·49) |
| Hamilton et al (2016)^29^ | QOF+ | Smoking status recorded | IMD | Possibly negative  [H/M,-] | Adj. OR for most vs least deprived (males):  pre-QOF+ = 0·99 (0·84-1·17)  post-QOF+ = 0·86 (0·74-0·99)  Adj. OR for most vs least deprived (females):  pre-QOF+ = 0·92 (0·80-1·36)  post-QOF+ = 0·77 (0·68-0·88) |
| Forster et al (2016)^28^ | NHS Health Check | Smoking status recorded | IMD | Possibly positive  [H,+] | Difference in % recorded for most vs least deprived:  males = Health Check = 0; controls = 4  females = Health Check = 0; controls = 1 |
| Access | | | | | |
| Blane et al (2017)^21^ | Routine care | Cessation advice | Carstairs | Possibly negative  [H/M,-] | Adj. OR for most vs least deprived = 0·80 (0·62-1·02) |
| Douglas & Szatkowski (2013)^27^ | Routine care | Cessation advice | Townsend/  Mosaic | Positive  [H/M,++] | Adj. OR for most vs least deprived (Townsend) = 1·28 (1·19-1·37)  Adj. OR for Mosaic G (social housing) vs A (professional) = 1·35 (1·20-1·52) |
| Hardy et al (2014)^30^ | Routine care | Cessation advice | Townsend | Positive  [H/M,++] | Adj. OR for most vs least deprived = 1·38 (1·14-1·68) |
| Taggar et al (2012)^12^ | QOF | Cessation advice | Townsend | Possibly positive  [H/M,+] | Adj. OR for most vs least deprived:  pre-QOF = 1·06 (0·90-1·26)  post-QOF = 1·20 (1·10-1·30) |
| Hamilton et al (2016)^29^ | QOF+ | Cessation advice | IMD | Mixed  [H/M,o]  [H/M,-] | Adj. OR for most vs least deprived (males):  pre-QOF+ = 1·05 (0·81-1·36)  post-QOF+ = 1·10 (0·81-1·48)  Adj. OR for most vs least deprived (females):  pre-QOF+ = 1·18 (0·84-1·65)  post-QOF+ = 1·01 (0·73-1·38) |
| Use | | | | | |
| Blane et al (2017)^21^ | Routine care | NRT prescription | Carstairs | Positive  [H/M,++] | Adj. OR for most vs least deprived = 1·41 (1·11-1·80) |
| Dhalwani et al (2014)^26^ | Routine care | NRT prescription | Townsend | Positive  [H,++] | Adj. OR for most vs least deprived = 1·29 (1·15-1·45) |
| Douglas & Szatkowski (2013)^27^ | Routine care | Cessation prescription | Townsend/  Mosaic | Positive  [H/M,++] | Adj. OR for most vs least deprived (Townsend) = 1·16 (1·05-1·28)  Adj. OR for Mosaic G (social housing) vs A (professional) = 1·50 (1·31-1·73) |

*Stop smoking services*

| Study | Intervention | Outcome measure | SES measure | Equity impact | Findings |
| --- | --- | --- | --- | --- | --- |
| Use | | | | | |
| DoH NI (2016)^34^ | SSS | Quit attempts | NIMDM | Possibly positive  [H,+] | No. quit attempts:  most deprived = 6824  least deprived = 1856 |
| ISD Scotland (2017)^37^ | SSS | Quit attempts | SIMD | Positive  [H,++] | Quit attempt rate among smokers:  most deprived = 7·4%  least deprived = 4·7% |
| NHS Digital (2017)^39^ | SSS | Quit attempts | Occupation | Possibly positive  [H,+] | No. quit attempts:  routine & manual = 99320  professional & managerial = 44429 |
| West et al (2013)^40^ | SSS | Quit attempts | Prescription exemption | Possibly positive  [M/L,+] | % of SSS clients in receipt of exemption: 44% in 2008/09 rising to 54% in 2010/11 |
| Brose et al (2013)^32^ | SSS | Medication type | Prescription exemption | Negative  [H/M,--] | Adj. OR for use of varenicline among those with exemption vs those that pay = 0·43 (0·42-0·44), p<0·001 |
| Brose & McEwen (2016)^33^ | SSS | Medication type | IMD | Negative  [H,--] | % use varenicline: most = 21·1; least = 30·6  % use NRT: most = 39·1; least = 28·1  p<0·001 |
| Hiscock et al (2015)^36^ | SSS | Medication type | Composite | Possibly negative  [H/M,-] | % clients who disadvantaged:  varenicline = 58·5 (50·8-66·4)  not varenicline = 66·3 (60·7-71·5) |
| Hiscock et al (2013)^35^ | SSS | Support type | IMD/  prescription | Unclear  [H/M,?] | Patterns of use similar across SES groups but descriptive analyses only |
| Hiscock et al (2015)^36^ | SSS | Support type | Composite | Possibly negative  [H/M,-] | % clients who disadvantaged:  group support = 57·5 (46·8-67·6)  drop-in support = 70·3 (63·7-76·2) |
| Success | | | | | |
| Bauld et al (2012)^31^ | SSS | Quit success | Composite | Negative  [H,--] | Adj. OR per unit increase in advantage = 1·33 (1·07-1·65) |
| Bauld et al (2016)^23^ | SSS | Quit success | Composite | Negative  [H,--] | Adj. OR for least vs most deprived = 1·4 (1·1-1·9) |
| Brose et al (2012)^22^ | SSS | Quit success | Occupation/  prescription/  IMD | Mixed  [H/M,--]  [H/M,--]  [H/M,o] | Adj. OR for occupation:  professional vs manual = 1·14 (1·06-1·23)  unemployed vs manual = 0·81 (0·75-0·87)  Adj. OR for prescription:  pay vs exempt = 1·20 (1·13-1·27)  Adj. OR per unit increase in IMD disadvantage:  0·98 (0·96-1·01), p=0·153 |
| Brose et al (2013)^32^ | SSS | Quit success | Prescription exemption | Negative  [H/M,--] | Adj. OR for exempt vs pay = 0·81 (0·79-0·87) |
| Brose & McEwen (2016)^33^ | SSS | Quit success | IMD/  prescription | Negative  [H,--] | Adj. OR per decile increase in IMD disadvantage = 0·968 (0·966-0·970)  Adj. OR for exempt vs pay = 0·840 (0·829-0·851) |
| DoH NI (2016)^34^ | SSS | Quit success | NIMDM | Negative  [H,--] | Quit success rate:  most deprived = 58·0%  least deprived = 61·2% |
| Hiscock et al (2013)^35^ | SSS | Quit success | Occupation/  prescription | Negative  [H/M,--] | Adj. OR for occupation:  professional vs manual = 1·17 (1·12-1·21)  unemployed vs manual = 0·71 (0·68-0·74)  Adj. OR for prescription:  pay vs exempt = 1·38 (1·35-1·42) |
| Hiscock et al (2015)^36^ | SSS | Quit success | Composite | Negative  [H/M,--] | Adj. OR for highest vs lowest SES = 1·44 (1·11-1·87) |
| ISD Scotland (2017)^37^ | SSS | Quit success | SIMD | Negative  [H,--] | Quit success rate (4 weeks):  most deprived = 35·7%  least deprived = 42·5%  Quit success rate (12 weeks):  most deprived = 20·4%  least deprived = 26·9% |
| McAlpine et al (2015)^38^ | SSS | Quit success | Occupation | Unclear  [L,?] | Quit success rate:  professional & managerial = 57%  routine & manual = 55%  [Descriptive analysis only] |
| NHS Digital (2017)^39^ | SSS | Quit success | Occupation/  prescription | Negative  [H,--] | Quit success rate by occupation:  routine & manual = 53·5%  professional & managerial = 56·8%  Quit success rate by prescription:  exempt = 48·8%  pay = 53·2% |

*Innovative interventions (6 studies of equity impact)*

| Study | Intervention | Outcome measure | SES measure | Equity impact | Findings |
| --- | --- | --- | --- | --- | --- |
| Access | | | | | |
| Gilbert et al (2017)^42^ | GP communication | SSS attendance | IMD | Possibly negative  [M,-] | OR for intervention vs control:  most deprived = 1·46 (1·02-2·07)  least deprived = 1·85 (1·09-3·14) |
| Venn et al (2016)^45^ | Mobile SSS | Register with MSSS | Occupation/  IMD/  prescription | Mixed  [M,++]  [M,o]  [M,o] | % clients from routine & manual groups:  MSSS = 33·3, standard = 27·2, p=0·002  Mean IMD of clients  MSSS = 39·5, standard = 39·7, p=0·77  % clients who pay for prescriptions:  MSSS = 23·0, standard = 22·3, p=0·71 |
| Use | | | | | |
| Turner et al (2013)^44^ | SSS relapse prevention medication | Accept NRT extension | Occupation/  prescription | Mixed  [M/L,+]  [M/L,--]  [M/L,-] | OR for accepting extended NRT course:  professional vs manual = 0·50 (0·11-2·22)  unemployed vs manual = 0·45 (0·22-0·91)  pay vs exempt = 2·84 (1·56-5·17) |
| Success | | | | | |
| Bennett et al (2015)^41^ | GP communication | Quit success | Reading level | Possibly positive  [M,+] | OR for intervention vs control:  easy reading = 1·50 (0·91-2·45)  standard reading = 1·05 (0·78-1·42) |
| Gilbert et al (2017)^42^ | GP communication | Quit success | IMD | Possibly negative  [M,-] | OR for intervention vs control:  most deprived = 1·18 (0·69-2·03)  least deprived = 1·51 (0·80-2·85) |
| Maskrey et al (2015)^43^ | SSS relapse prevention booklet | Quit success | Education/  prescription | Unclear  [H/M,?] | No difference between intervention & control; interaction with SES (both measures) not significant |
| Stapleton et al (2013)^24^ | Bupropion vs NRT vs combination | Quit success | Education/  benefits | Unclear  [L,?] | No difference between treatments; interaction with SES (both measures) not significant |
| Venn et al (2016)^45^ | Mobile SSS | Quit success | Occupation | Possibly positive  [M,+] | % MSSS clients who quit at 4 weeks:  routine & manual = 24·5%  all clients = 18·3%  % standard service clients who quit at 4 weeks:  routine & manual = 33·2%  all clients = 33·5% |

*Innovative interventions (4 studies targeted at disadvantaged groups)*

| Study | Intervention | Outcome measure | Intervention effectiveness | Findings |
| --- | --- | --- | --- | --- |
| Use | | | | |
| Ormston et al (2015)^47^ | Incentives (mixed settings) | Quit attempts | Unclear  [H/M,?] | No. quit attempts increased by 44% in most deprived areas of Tayside (with quit4u accounting for 35·5% of attempts) but similar increases seen across Scotland |
| Radley et al (2013)^48^ | Incentives (pharmacy) | Quit attempts | Positive  [M/L,++] | RR for GIUFB vs non-incentive Scottish SSSs = 1·58 (1·38-1·81) |
| Tappin et al (2015)^49^ | Incentives (SSS) | Quit attempts | Neutral  [H/M,o] | RR for intervention vs control = 1·04 (0·96-1·13), p=0·37 |
| Success | | | | |
| Kassim et al (2016)^46^ | Community outreach SSS | Quit success | Possibly positive  [M,+] | Intervention quit success at 4 weeks: 68·8% (64-74) compared to 36% for all SSS clients from same area |
| Ormston et al (2015)^47^ | Incentives (mixed settings) | Quit success | Positive  [H/M,++] | Quit success at 1 month: quit4u = 49·4%; non-quit4u = 33·7%; relative difference = 1·479 (1·404-1·558)  Quit success at 3 months: quit4u = 30·7%; non-quit4u = 14·2%; relative difference = 2·158 (1·985-2·347)  Quit success at 12 months: quit4u = 9·3%; non-quit4u = 6·5%; relative difference = 1·443 (1·132-1·839) |
| Radley et al (2013)^48^ | Incentives (pharmacy) | Quit success | Positive  [M/L,++] | Quit success at 4 weeks: RR for GIUFB vs non-incentive Scottish SSSs = 2·03 (1·60-2·59)  Quit success at 12 weeks: GIUFB = 31·.8% vs 15% for all Scottish SSSs |
| Tappin et al (2015)^49^ | Incentives (SSS) | Quit success | Positive  [H/M,++] | Quit success at 8 weeks: intervention = 22·5%; control = 8·6%; RR=2·63 (1·73-4·01)  Quit success at 6 months post-partum: intervention = 15%; control = 4%; RR=3·88 (2·41-6·23) |

1. **National equity impact estimates: methods & sensitivity analyses**

| *Data sources used to estimate total smoker population* |
| --- |
| England |
| Annual national mid-year population estimates (ONS, 2017a) |
| Census data on proportion of population falling into each NS-SEC occupation group (NOMIS, 2017) |
| Annual Population Survey estimates of smoking prevalence by NS-SEC occupation group (ONS, 2017b) |
| Scotland |
| Estimated number of smokers by SIMD (ISD Scotland, 2017) |
| Northern Ireland |
| Annual national mid-year population estimates by super output area (NISRA, 2016) |
| NIMDM deprivation rankings by super output area (NISRA, 2017) |
| NI Health Survey estimates of smoking prevalence by NIMDM (Corrigan & Scarlett, 2017) |

Corrigan D, Scarlett M (2017). *Health Survey (NI) 2016/17*. Belfast: Information Analysis Directorate. <https://www.health-ni.gov.uk/publications/tables-health-survey-northern-ireland>

ISD Scotland (2017). *NHS Smoking Cessation Service Statistics (Scotland) 1^st^ April 2016 to 31^st^ March 2017*. Edinburgh: ISD Scotland. <http://www.scotpho.org.uk/publications/reports-and-papers/nhs-smoking-cessation-service-statistics-scotland-1st-april-2016-to-31st-march-2017/>

# Nomis (2017). *2011 Census Data on Nomis*. Durham: ONS. <https://www.nomisweb.co.uk/census/2011>

# Northern Ireland Statistics and Research Agency (2016). *2016 Mid Year Population Estimates for Northern Ireland*. Belfast: NISRA. <https://www.nisra.gov.uk/publications/2016-mid-year-population-estimates-northern-ireland>

Northern Ireland Statistics and Research Agency (2017). *Northern Ireland Multiple Deprivation Measures 2017: SOA level results*. Belfast: NISRA. <https://www.nisra.gov.uk/publications/nimdm17-soa-level-results>

# Office for National Statistics (2017a). *Population Estimates for UK, England and Wales, Scotland and Northern Ireland*. Titchfield: ONS. <https://www.ons.gov.uk/peoplepopulationandcommunity/populationandmigration/populationestimates/datasets/populationestimatesforukenglandandwalesscotlandandnorthernireland>

Office for National Statistics (2017b). *Smoking prevalence by NS-SEC using the Annual Population Survey by sex and age, England, 2012 to 2016*. Newport: ONS. <https://www.ons.gov.uk/peoplepopulationandcommunity/healthandsocialcare/drugusealcoholandsmoking/adhocs/007705smokingprevalencebynssecusingtheannualpopulationsurveybysexandageengland2012to2016>

| *Comparison of NS-SEC occupation coding across English data sources* | |
| --- | --- |
| Population estimates/census/smoking prevalence | SSS national reports |
| Managerial & professional occupations | Managerial & professional occupations |
| Intermediate occupations | Intermediate occupations |
| Routine & manual occupations | Routine & manual occupations |
| Never worked, unemployed & not elsewhere classified | Never worked or unemployed for over 1 year |
|  | Full-time students |
| Allocated to one of above categories based on last occupation | Retired |
|  | Home carers (unpaid) |
|  | Prisoners |
|  | Sick/disabled & unable to return to work |
|  | Unable to code |

*Availability of SES data in national SSS reports*

|  | England | | | Northern Ireland | | | Scotland | | |
| --- | --- | --- | --- | --- | --- | --- | --- | --- | --- |
|  | No. quit attempts | Unable to  code SES^(i)^ | | No. quit attempts | SES  missing | | No. quit attempts | SES  missing | |
|  |  | No. | % |  | No. | % |  | No. | % |
| 2012/13 | 724247 | 268980 | 37·1 | 31987 | 4874 | 15·2 | 116577 | 363 | 0·31 |
| 2013/14 | 584914 | 229679 | 39·3 | 26273 | 3291 | 12·5 | 94016 | 173 | 0·18 |
| 2014/15 | 450582 | 175050 | 38·8 | 21382 | 813 | 3·8 | 67940 | 227 | 0·33 |
| 2015/16 | 382500 | 142286 | 37·2 | 20987 | 175 | 0·8 | 64838 | 37 | 0·06 |
| 2016/17 | 307507 | 115878 | 37·7 | n/a | n/a | n/a | 59767 | 41 | 0·07 |

1. SES data either missing or could not be mapped to one of occupational groups used in estimating total smoker population due to inconsistencies in the coding systems adopted

*Definitions & methods used in sensitivity analyses*

- Equity impact = difference in quit attempt (or success) rate between lowest & highest SES group
- For England: lowest SES = routine & manual occupations; highest SES = managerial & professional occupations
- For Northern Ireland: lowest SES = NIMDM quintile 1; highest SES = NIMDM quintile 5
- Range of possible values for equity impact calculated by treating cases with missing/uncodeable SES as follows:

Allocate missing cases to lowest SES group

Exclude missing cases from analysis

Allocate missing cases to highest SES group

*Sensitivity analyses for equity impact of English SSSs*


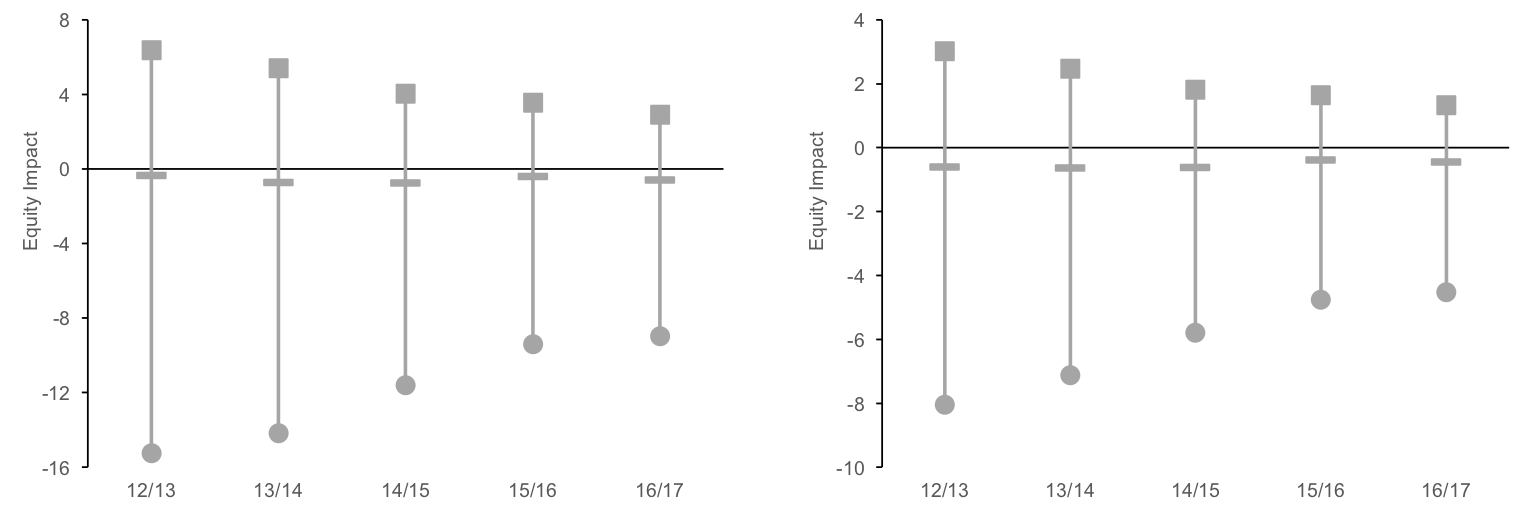


(b) Quit success rate

(a) Quit attempt rate

*Sensitivity analyses for equity impact of Northern Ireland SSSs*


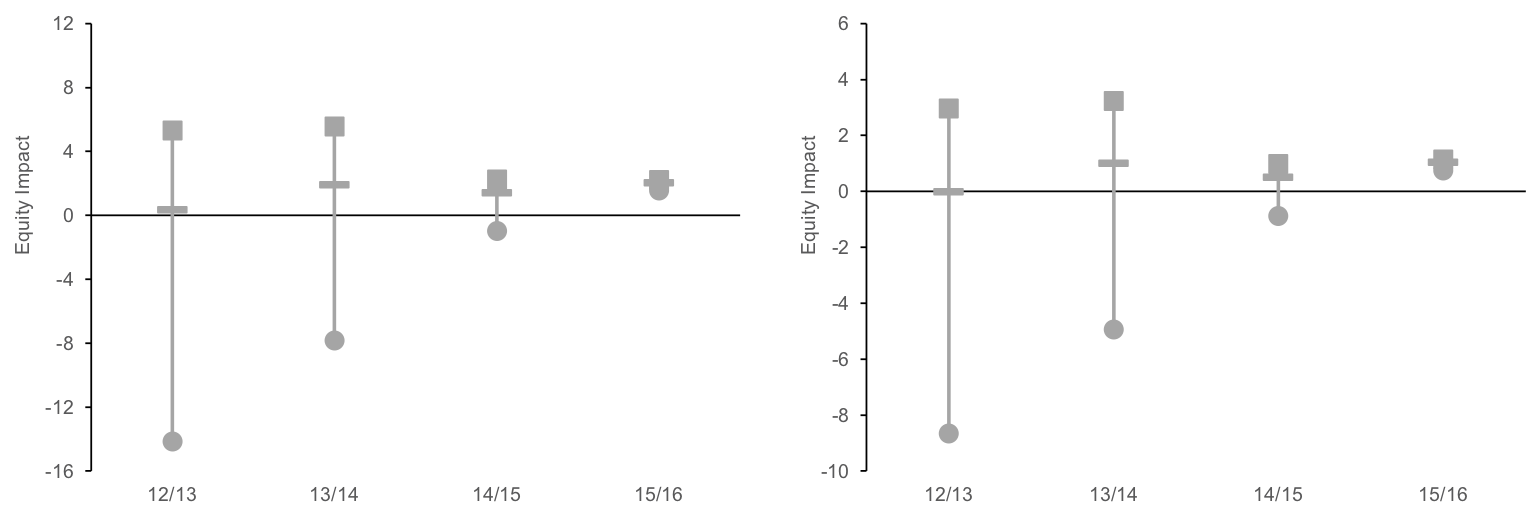


(b) Quit success rate

(a) Quit attempt rate
